# A 3-miRNA signature predicts prognosis of pediatric and adolescent cytogenetically normal acute myeloid leukemia

**DOI:** 10.18632/oncotarget.17151

**Published:** 2017-04-17

**Authors:** Ruiqi Zhu, Weiwei Zhao, Fengjuan Fan, Liang Tang, Jingdi Liu, Ting Luo, Jun Deng, Yu Hu

**Affiliations:** ^1^ Institute of Hematology, Union Hospital, Tongji Medical College, Huazhong University of Science and Technology, Wuhan, 430030, China; ^2^ Department of Epidemiology and Biostatistics, School of Public Health, Harbin Medical University, Harbin, 150086, China

**Keywords:** pediatric and adolescent, CN-AML, miRNA, signature, prognosis

## Abstract

Acute myeloid leukemia is a hematologic malignancy with significant molecular heterogeneity. MicroRNAs have important biological functions and play critical roles in pathogenesis and prognosis in a variety of cancers including acute myeloid leukemia. Some reports have constructed risk stratification systems for adult acute myeloid leukemia patients using microRNAs to predict an optimal outcome of patients. However, little has been done in pediatric and adolescent patients. The purpose of this study is to identify a panel of microRNA signature that could predict prognosis in younger cytogenetically normal acute myeloid leukemia patients by analyzing the data from The Cancer Genome Atlas. A total of 59 cytogenetically normal acute myeloid leukemia patients under 21 years with corresponding clinical data were enrolled in our study. Using univariate Cox's model, we found 17 miRNAs were significantly related with overall survival in pediatric and adolescent cytogenetically normal acute myeloid leukemia patients but no clinical parameter was found significant related with overall survival. The multivariate Cox regression identified high expression of hsa-miR-146b was independent poor prognostic factor and high expression of hsa-miR-181c and hsa-miR-4786 appeared to be favorable factors. A model was proposed based on these three miRNAs. Leave-one-out Cross Validation method and Permutation Test was further used to evaluate this model. The function role of has-mir-181c was further studied by carrying out flow cytometry and cell counting kit-8 (CCK-8) in U937 cell line. The results indicate that the 3-microRNA-based signature is a reliable prognostic biomarker for pediatric and adolescent cytogenetically normal acute myeloid leukemia patients.

## INTRODUCTION

Pediatric acute myeloid leukemia (AML) is a rare and heterogeneous childhood cancer, with an incidence of approximately 7 cases per million children annually [1]. Although board overlap lies between AML for pediatric patients, adolescents and adults in diagnosis, treatment and prognosis, differences still exist. Cytogenetically normal acute myeloid leukemia (CN-AML) is a kind of AML that no cytogenetic abnormality is detectable in leukemic cells. In pediatric CN-AML patients, outcome of the patients is associated with other co-occurring mutations. CN-AML without any molecular mutation is considered to be at intermediate risk. When harboring NPM1 or CEBPA double mutation, pediatric CN-AML patients are considered favorable prognosis. In contrast, a FLT3-ITD mutation to wild-type ratio (ITD allelic ratio) of > 0.4 is associated with adverse outcome [2]. MicroRNAs (miRNAs) are small non-coding RNA molecules that can regulate gene expression at the post-transcriptional level. MiRNAs are enrolled in tumorigenesis of acute myeloid leukemia and impact hematopoietic cell differentiation, proliferation and treatment response. Emerging evidence suggest miRNAs expression signatures can also predict outcomes of various human cancers [3–6], including hematological malignancies such as adult AML[7] and primary plasma cell leukemia [8]. However, there were few studies investigated the prognostic value of miRNAs in pediatric and adolescent AML patients. Whether miRNA-expression profiling can identify different outcomes of various molecular mutation remains unclear. Here we conducted a study using the dataset extracted from The Cancer Genome Atlas (TCGA, https://cancergenome.nih.gov/) and constructed a 3-miRNA signature which may be used to predict the outcome of pediatric and adolescent CN-AML patients.

## RESULTS

### Identification of miRNAs associated with outcome

A total of 301 patients with microRNA information were selected, 59 patients who are cytogenetically normal were enrolled in our study. MicroRNAs were transformed from continuous variable into binary variable—high miRNA expression (expression level greater than the median) status equals 1 and low status equals 0. A total of 549 miRNAs were selected for further analysis from 1539 miRNAs according to the exclusion criteria. After univariate cox's model filtering, 17 miRNAs were suggested to be correlated with prognosis (Table 1).

**Table 1:** Univariate Cox analysis of 549 miRNAs

MicroRNA	*P*-value	FDR	Type
hsa.mir.181c	0.000	0.001	Risky
hsa.mir.146b	0.000	0.001	Protective
hsa.mir.153.1	0.003	0.006	Risky
hsa.mir.500a	0.007	0.011	Protective
hsa.mir.501	0.012	0.017	Protective
hsa.mir.181d	0.014	0.020	Risky
hsa.mir.3174	0.018	0.024	Risky
hsa.mir.30b	0.018	0.024	Risky
hsa.mir.3176	0.019	0.025	Risky
hsa.mir.570	0.022	0.028	Risky
hsa.mir.4484	0.023	0.029	Risky
hsa.mir.378d.2	0.026	0.032	Risky
hsa.mir.125b.1	0.027	0.034	Risky
hsa.mir.4786	0.028	0.034	Risky
hsa.mir.500b	0.031	0.038	Protective
hsa.mir.30d	0.032	0.038	Risky
hsa.mir.548s	0.032	0.039	Risky

### Construction of miRNA signature

By carrying out multivariate cox regression formula, we identified 3 miRNAs (mir-181c, mir-146b and mir-4786) that were independently associated with prognosis. High expression of hsa-miR-146b was independent poor prognostic factor and high expression of hsa-miR-181c and hsa-miR-4786 appeared to be favorable factors. Then the risk score was calculated through the three miRNA status and their weight on OS, which is represented by the β coefficient in multivariate cox model. The risk score = (1.652* status of has-mir-146b)–(1.838* status of hsa-mir-181c)–(1.455* status of has-mir-4786). Next, risk score was calculated in each of the 59 patients and the ones whose risk scores greater than the median were assigned to high risk group and the others belong to low risk group.

### Clinical and molecular characteristics of patients associated with 3 miRNAs and the risk score

Clinical characteristics and molecular alteration information of the patients with the risk score were displayed in Tables 2, 3, 4. Clinical characteristics were utilized to fit the Univariate Cox's model and the results were shown in Table 5. In our study, age at diagnosis, gender, WBC at diagnosis, bone marrow blasts and peripheral blasts did not show significance with prognosis. NPM1 and CEBPA mutations are correlated with favorable prognosis, with *P-value* of 0.031 and 0.05, respectively.

**Table 2:** Correlation between miRNA score and clinical and laboratory features in pediatric and adolescent CN-AML patients (*n* = 59)

Characteristics	Total	microRNA score		*P*-value
		Low (*n* = 30)	High (*n* = 29)	
Age (Mean ± SD, years)	11.5 ± 5.2	12.0 ± 4.7	10.9 ± 5.7	0.449
Male sex-no. (%)	36 (61.0%)	17 (56.7%)	19 (65.5%)	
WBC at diagnosis(10^−9^/liter)*	69.9 (0.9–446)	67.3 (5.1–210)	72.5 (0.9–446)	0.807
Bone marrow blasts (%)	71.3 (21-98)	74.8 (21-98)	67.5 (25–96)	0.168
Peripheral blasts (%)	55.2 (0–97)	67.1 (18.5–97)	42.3 (0–95)	0.002
FAB Category				
M0	1 (1.7%)	0	1 (3.4%)	NA
M1	16 (27.1%)	12 (40.0%)	4 (13.8%)	**0.049**
M2	14 (23.7%)	8 (26.7%)	6 (20.7%)	0.815
M4	13 (22.0%)	5 (16.7%)	8 (27.6%)	0.486
M5	4 (6.8%)	2 (6.7%)	2 (6.9%)	> 0.999
NOS	7 (11.9%)	2 (6.7%)	5 (17.2%)	0.394
Unknown	4 (6.8%)	1 (3.3%)	3 (10.3%)	0.580

*WBC at diagnosis: White Blood Cells at dignosis.

**Table 3:** Correlation between miRNA score and other gene alterations

Mutation	Total	microRNA score		*P*-value
		Low (*n* = 30)	High (*n* = 29)	
FLT3-ITD allelic ratio > 0.4	14 (23.7%)	5 (13.8%)	9 (33.3%)	0.322
NPM1	15 (25.4%)	12 (37.9%)	3 (13.3%)	0.021
CEBPA	14 (23.7%)	12 (41.3%)	2 (6.7%)	0.007

**Table 4:** Association between molecular mutations and FAB category with 3 miRNAs

Variants	hsa-miR-181c			hsa-miR-146b			hsa-miR-4786		
	High (29)	Low (29)	*P*-value	High (29)	Low (20)	*P*-value	High(29)	Low (30)	*P*-value
NPM1	9	6	0.500	7	8	> 0.999	9	6	0.500
CEBPA	11	3	**0.027**	1	13	**0.001**	9	5	0.322
FLT3-ITD	9	13	0.479	16	6	**0.012**	11	11	> 0.999
FAB									
M0	0	1	> 0.999	1	0	> 0.999	0	1	> 0.999
M1	13	3	**0.007**	7	9	0.831	7	9	0.839
M2	10	4	0.109	6	8	0.815	8	6	0.705
M4	3	10	0.069	6	7	> 0.999	7	6	0.945
M5	0	4	0.129	1	3	0.629	3	1	0.580
NOS	2	5	0.449	5	2	0.394	4	3	0.962
Unknown	1	3	0.629	3	1	0.580	0	4	0.129

**Table 5:** Univariate Cox analysis of clinical parameters with the prognosis

Variants	*P*-value
Age at diagnosis	0.249
Gender	0.543
WBC at diagnosis	0.533
Bone marrow blasts	0.733
Peripheral blasts	0.406
FLT3-ITD positive	0.153
NPM1 mutation	0.031
CEBPA mutation	0.050

### MiRNA signature was an independent prognosis predictor of pediatric and adolescent CN-AML

After adjusting for NPM1 and CEBPA mutations, the 3-miRNA also independently predicted OS (*P* = 0.003) and DFS (*P* = 0.000).

### Correlation of microRNA prognostic signature with clinical or laboratory features and gene alterations

Higher risk score is associated with lower percentage of peripheral blasts in laboratory features. In contrast, no association was found between risk score and clinical features (Table 1). Genetic alterations were found different between high and low risk score groups: Patients with lower scores more often had NPM1 (*P* = 0.021) and CEBPA mutations (*P* = 0.007), which means patients with lower risk score more likely had favorable mutations (Table 2). High expression level of miR-181c (*P* = 0.027) and low expression level of miR-146b (*P* = 0.001) were more often had CEBPA mutations. High expression level of miR-146b (*P* = 0.012) more often had FLT3-ITD mutations. Association between miRNAs, the risk score and FAB category were also assessed. High expression level of miR-181c and lower risk score more often had M1 subtype of leukemia. In our study, no association was found between FLT-ITD allelic ratio > 0.4 and the microRNA signature.

### Performance of miRNA signature

**Figure 1:**
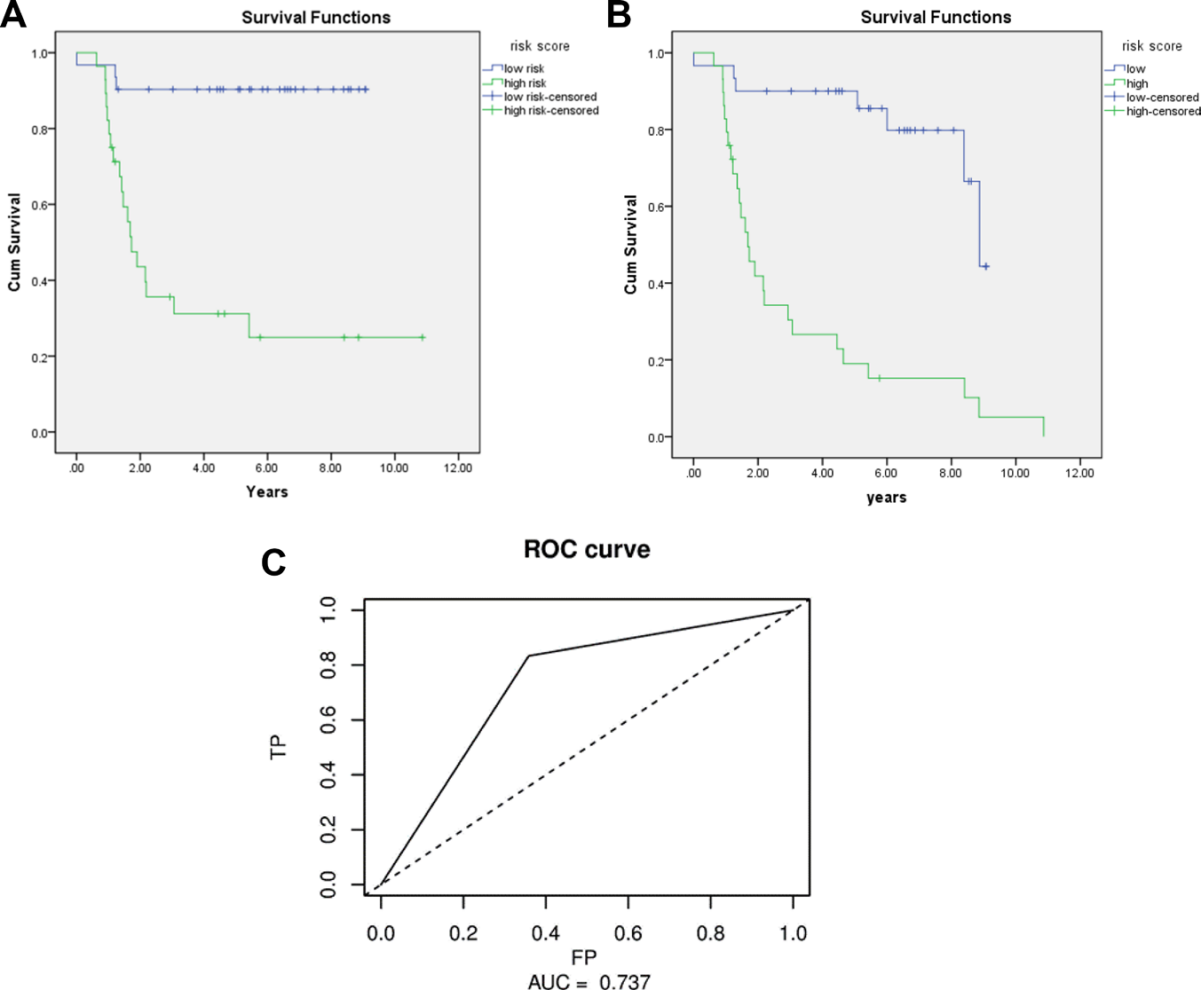
Kaplan-Meier for OS/DFS in low risk and high risk group and AUC curve for the risk score

**Figure 2:**
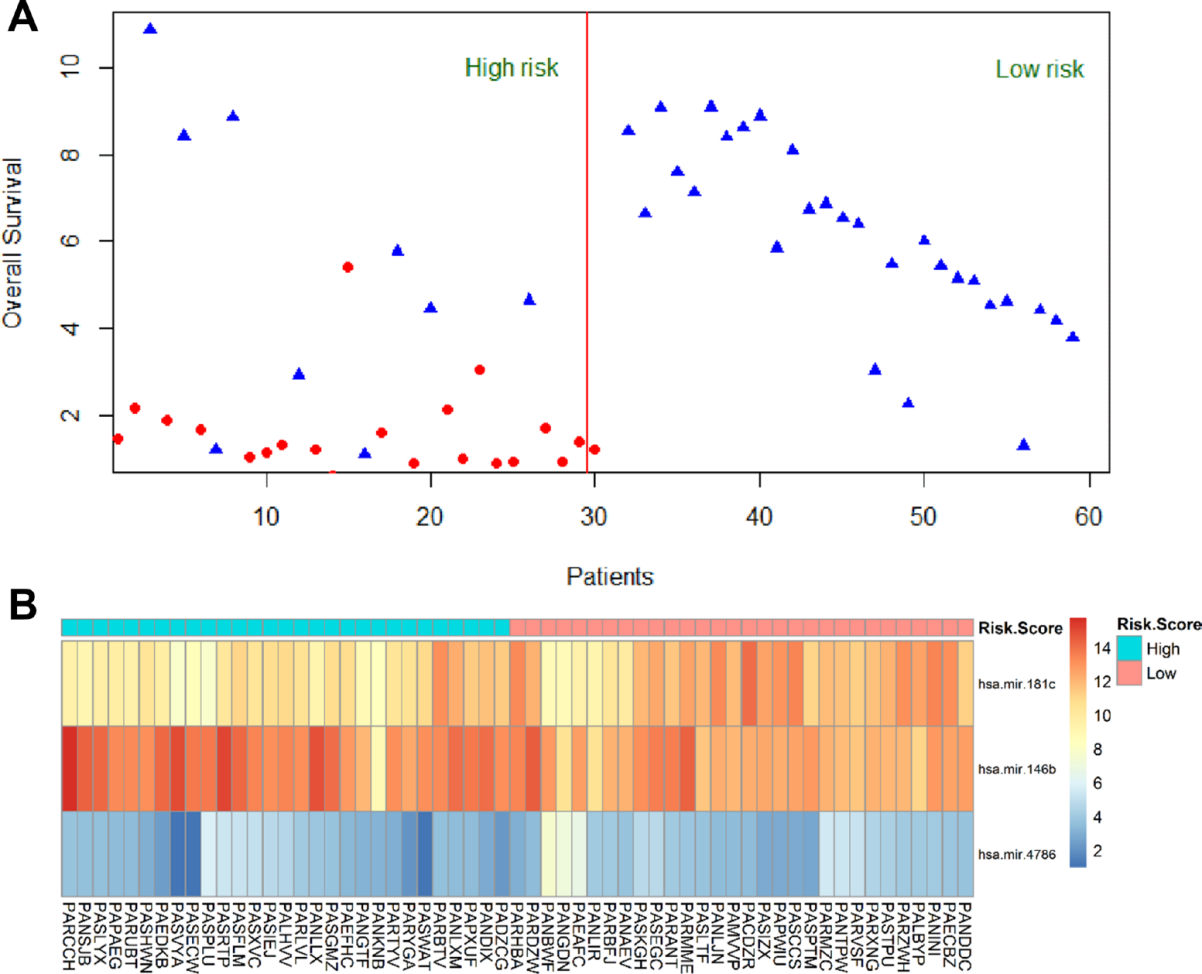
Heatmap for 3 miRNAs expression level and survival status in all 59 patients

The Kaplan-Meier curve was applied to the 3-miRNA signature using the group separation of risk score. The results showed that patients in the high risk score group had significant worse OS/DFS than those in low risk score group (*P* = 0.000) (Figure 1A, 1C). The AUC of signature was 0.737 (Figure 1B). A figure and heatmap were created to evaluate the signature, and it was clearly shown that most dead patients were in high risk group and suffered worse OS (Figure 2). The results confirm that the 3-miRNA signature has the power to differentiate patients into high-risk and low-risk groups.

### Permutation test and leave-one-out cross validation

**Figure 3:**
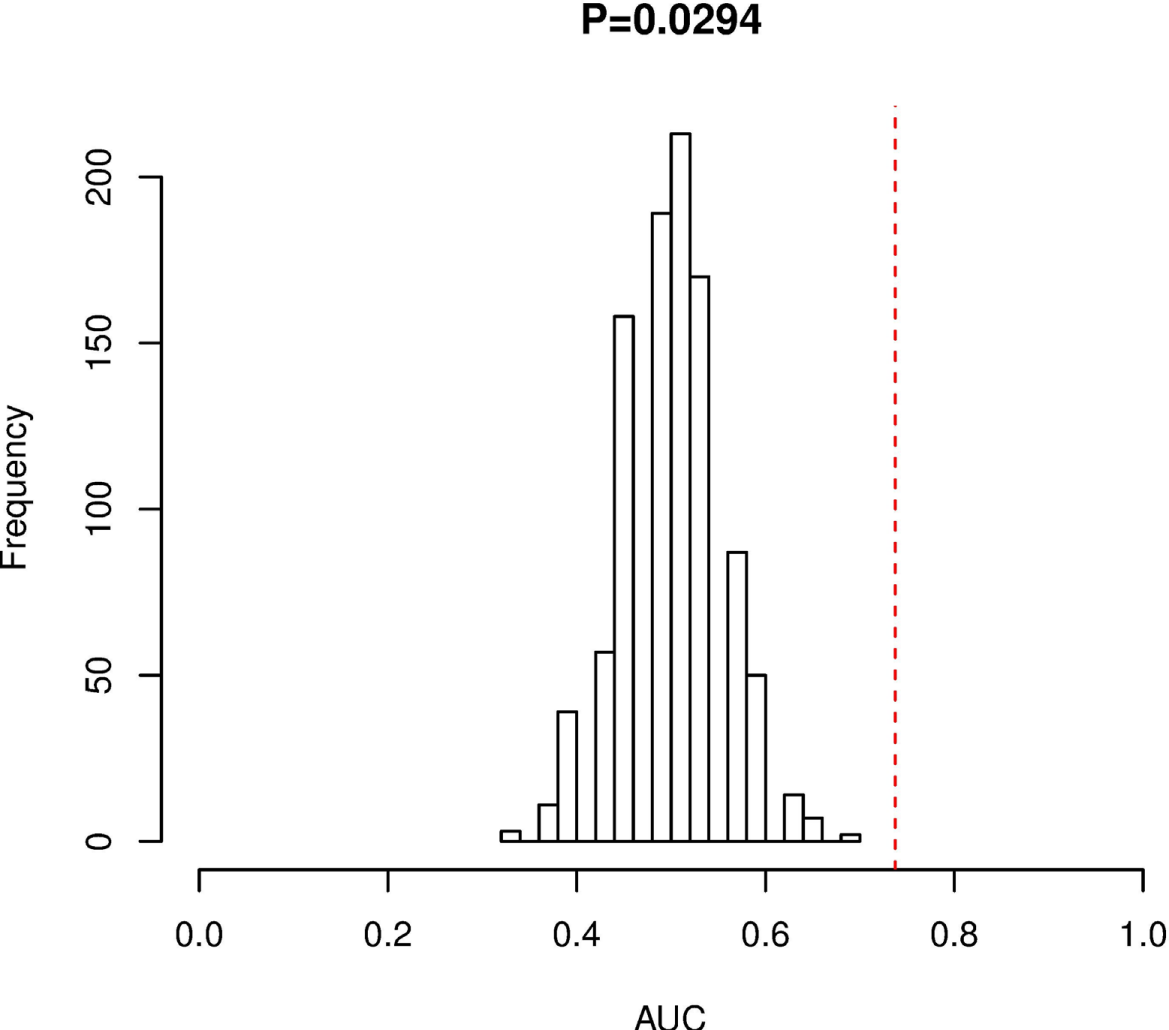
Permutation test for 3-miRNA signature

In order to validate whether the 3-miRNA signature is able to applied to other pediatric and adolescent CN-AML patients, we did permutation test and found that the AUC of random systems showed great significance with that of our studied cohort (*P-value* = 0.029) (Figure 3). These results indicate that our model could successfully predict the prognosis of pediatric and adolescent CN-AML patients. LOO-CV showed an AUC of 0.69 which also validates the 3-miRNA signature performs well.

### Bioinformatic analysis of target genes and pathways

Target genes of mir-181c, mir-146b and mir-4786 were predicted by three prediction tools—TargetScan, miRanda and miRTarBase. Considering false positive of prediction tools, TargetScan and miRanda were used to predict target genes of miR-181c and miR-146b, while TargetScan and miRTarBase were applied for miR-4786. MiRanda did not provide data of miR-4786. 327 target genes predicted by both tools were extracted (Supplementary Table 1). Part of the results were shown in Table 6. KEGG and part of GO results were displayed in Table 7 and Table 8. More GO results were shown in Supplementary Table 2.

**Table 6:** Part of target genes of three miRNAs

	Target		Target		Target
	ZNF667		NOVA1		YWHAE
	RLF		TRAF6		MORN4
	C16orf87		CD80		TTPAL
	C7orf41		NRAS		APP
	BEND3		FAM26E		APP
			E		
	LRRC8D		SCN3B		RNF115
	PLCL2		GOSR1		CA6
hsa-mir-181c	SPP1	hsa-mir-146b	ZNF148	hsa-mir-4786	IL17REL
	HMGB2		SIAH2		TBC1D15
	LIN28A		MMP16		COX6A1
	FLT1		ZNRF3		UBE2D3
	BAG4		POU3F2		COX6A1P2
			2		
	IL1A		ROBO1		LRTOMT
	RABGEF1		WASF3		TCEB3
	1				
	FIGN		FZD1		TRIM17

**Table 7:** GO analysis results of 352 targets

Category	ID	Term	Counts	*P*-Value
Biological process	0045449	Regulation of transcription	64	0.002
	0006355	Regulation of transcription, DNA-dependent	47	0.003
	0051252	Regulation of RNA metabolic process	47	0.004
	0045664	Regulation of neuron differentiation	8	0.008
Cellular components	0031974	Membrane-enclosed lumen	49	0.000
	0043233	Organelle lumen	48	0.000
	0031981	Nuclear lumen	40	0.000
	0070013	Intracellular organelle lumen	45	0.000
	0005654	Nucleoplasm	26	0.002
	0044451	Nucleoplasm part	18	0.004
Molecular function	0008270	Zinc ion binding	61	0.002

(*P* = 0.01).

**Table 8:** KEGG pathway results

Term	ID	*P*-Value	Gene
Alanine, aspartate and glutamate metabolism	00250	0.009796	GFPT1, AGXT, AGXT2, RIMKLB
Vitamin B6 metabolism	00750	0.012	PDXK, PNPO
Hippo signaling pathway -multiple species	04392	0.0314	RASSF6, MOB1B, RASSF1
Acute myeloid leukemia	05221	0.043011	NRAS, RUNX1, MAPK1
Prion diseases	05020	0.048606	IL1A, MAPK1, EGR1

(*P* = 0.05).

### MiR-181c mimics treatment in U937 cells

**Figure 4:**
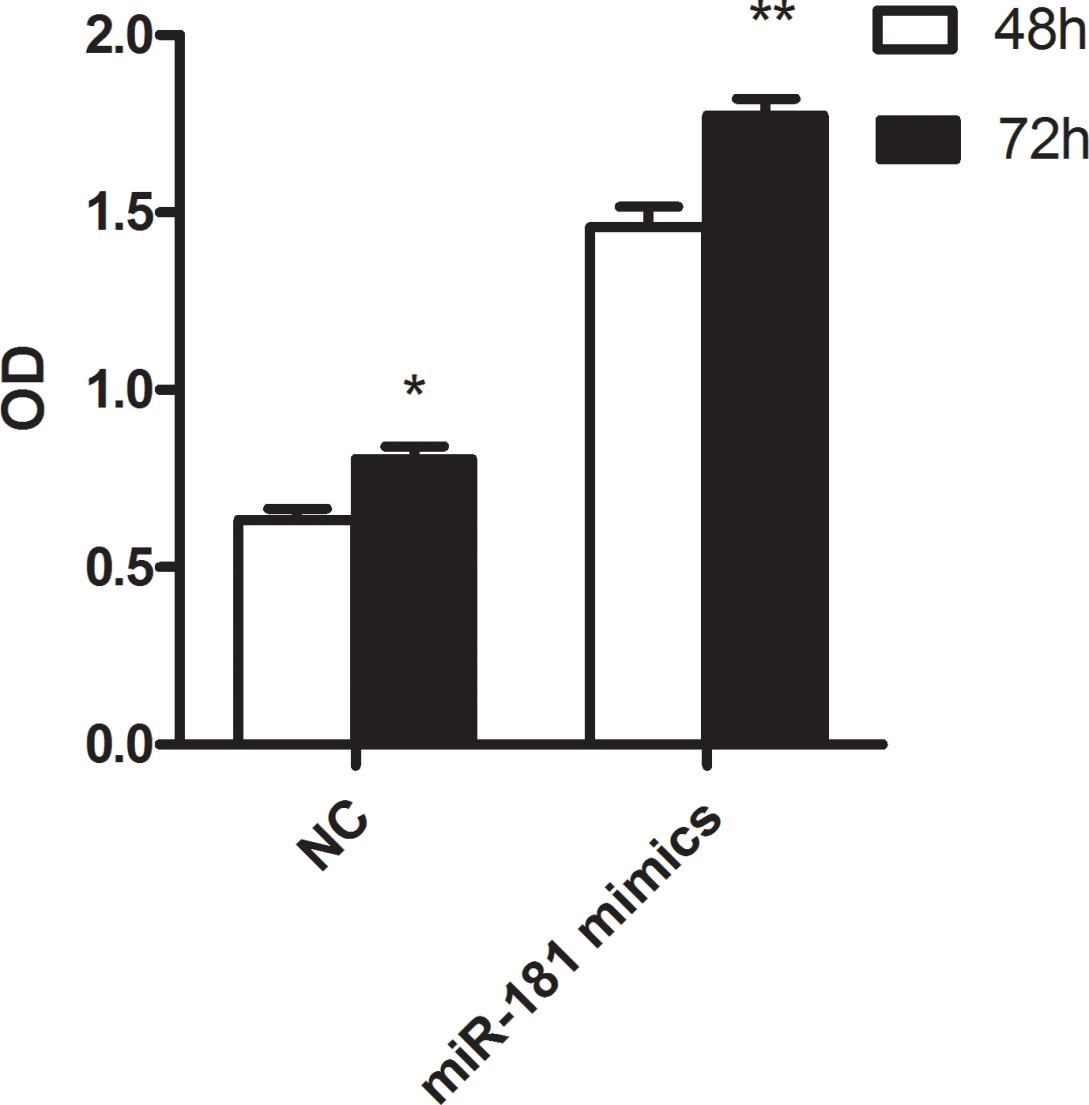
The result of cell proliferation in U937 cell line treated with miR-181c mimics and NC

**Figure 5:**
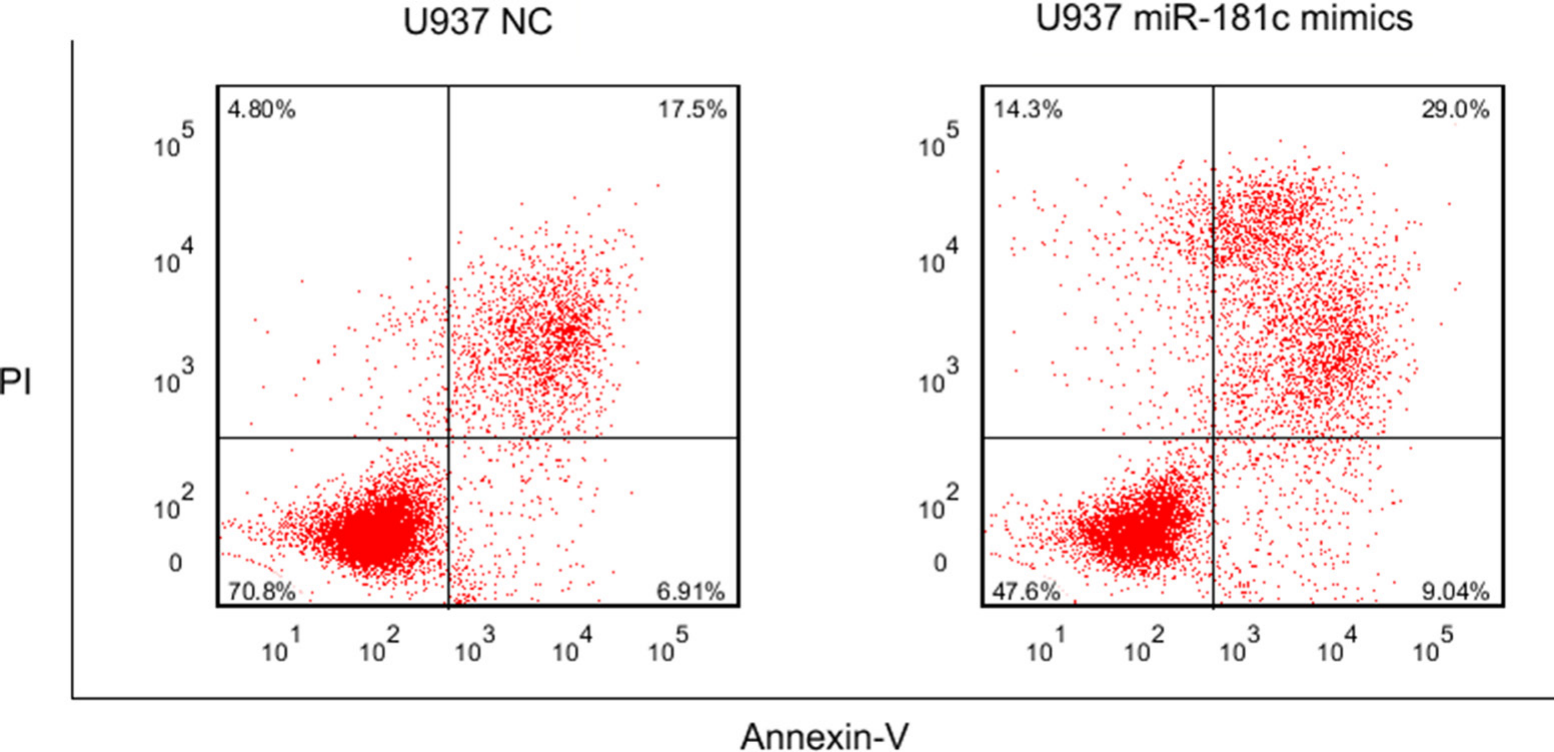
The result of apoptosis in U937 cell line treated with miR-181c mimics and NC

The functional roles of these miRNAs needs to be further studied. We focused on miR-181c, whose family members were broadly reported in AML including cytogenetically normal subtype. U937 was commonly regarded as cytogenetically normal AML cells and they were used in our study. After 48 and 72 hours transfection with miR-181c mimics and negative control, the proliferation activity was significantly lower in group treated with miR-181c mimics (*P* = 0.018 and 0.004, respectively.) (Figure 4). Flow cytometry was further carried out to observe apoptosis between miR-181c treatment and NC group. The result shows that miR-181c also exhibits ability of inducing apoptosis significantly in U937 cells (Figure 5).

## DISCUSSION

Ample evidence suggests microRNAs play important roles in progress and prognosis in pediatric and adolescent AML. Pediatric AML patients always carry cytogenetically abnormalities and the abnormal karyotypes rate reaches 70–80% compared to 55% in adult AML patients [2]. Little has been done to construct a miRNA signature scoring system to accurately predict the outcome of pediatric and adolescent CN-AML patients.

Here we used pediatric and adolescent TCGA AML dataset to construct a 3-miRNA signature (mir-181c, mir-146b and mir-4786) which underwent rounds of statistical analysis to ensure the strong and independent influence of outcome built for predicting and further exploring pediatric CN-AML molecular mechanisms .

CN-AML patients are considered to be an intermediate-risk prognosis category, and prognosis can be further divided into subgroups based on favorable (NPM1, CEBPA) or unfavorable (FLT3-ITD) genetic mutations. In our study, CEBPA and NPM1 mutations are not only predict favorable prognosis of pediatric CN-AML patients (CEBPA, *P* = 0.05, NPM1, *P* = 0.031), but also correlate with the risk score—lower risk score patients are more likely to have longer OS and DFS, and more often had these favorable mutations correlated with favorable outcomes. Lower expression level of aggressive miR-146b was more often had favorable CEBPA mutations and less often aggressive FLT3-ITD mutations. The condition was contrary in miR-181c. MiR-181 family (including miR-181c) has also been reported to be upregulated in patients with CEBPA mutations [9]. Our findings are consistent with the previous study.

However, FLT3-ITD neither shows relationship with the prognosis nor the 3-miRNA signature. One reason may be the small sample in our study cannot draw a significant conclusion statistically. Another reason may be 10 patients with FLT3-ITD mutations in our study also had NPM1 (8 patients) or CEBPA (2 patients) mutations and 8 of these patients are alive during the follow-up period. NPM1 and CEBPA are reported a positive effect on survival irrespective of FLT3-ITD mutations in some studies [9–11]. Therefore, FLT3-ITD did not present its predictive role in our study.

Over expression of miR-181 family was also reported associated with erythroid differentiation and monocytic differentiation, which means they potentially be involved in the differentiation block of M1 blasts [9, 12]. This may explain why higher expression level of miR-181c more often had M1 subtype of leukemia in our study. Lower risk score also more often possessed M1 subtype, which was mostly ascribed to miR-181c. MiR-181 family members were found downregulated in unfavorable adult hematologic malignancies [9, 13–16]. It has been reported that they increase expression of a series of homeobox superfamily genes (i.e., HOXA7, HOXA9, HOXA11 and PBX3) [15]. Homeobox superfamily genes are highly conserved and play important roles in a variety kinds of biological processes including apoptosis, differentiation and tumorigenesis, acting as tumor suppressors [17]. MicroRNA 181 family (including miR-181c) may act as a protective factor through HOX in pediatric and adolescent leukemia patients, our functional study in U937 cells consists with these findings. Su et al. suggest MiR-181 family (including miR-181c) expression level is elevated in adult acute myeloid leukemia patients compared with normal population and miR-181 family induces acute leukemogenesis through hindering granulocytic and macrophage-like differentiation in acute myeloid leukemia cell lines. However, the relationship between prognosis and miR-181 family expression level was not executed in this study [18]. More studies should be done to clarify relations between miR-181 family and pediatric acute myeloid leukemia progress and prognosis. MiR-146b was also reported to be a differential expression miRNA between M1 and M5 FAB subtypes, and was speculated participating in the block of M1 development and maturation, although its further function was not studied in AML [12]. It was found to play an oncogenic role in hematologic malignancies including T-ALL and lymphoma [19, 20], and to promote leukemic transformation of hematopoietic cells within BCR-ABL1-positive microvesicles [21]. MiR-146b is a key regulator accelerated the transformation by targeting NUMB and other genes, causing genome instability [20]. MiR-146b has been reported in a variety of kinds of cancer, acting as tumor promotor [22, 23] or suppresser [24–26] depending on different cancer types. In contrast, little was reported about mir-4786.

To gain a further insight into the functional role of the 3-miRNA signature, we extracted their target genes and carried out GO and KEGG pathway analysis. Interestingly, several pathways have compact relation with progression and prognosis of acute myeloid leukemia. For example, glutamate metabolism has critical role in AML progression. AML cells require glutamine to adapt to increased biosynthetic activity, and glutaminolysis inhibition activated mitochondrial apoptosis and provides a novel therapeutic strategy for AML [27]. In addition, vitamin B6 participates in many biological processes and is suggested to be related to carcinogenesis and tumor growth [28, 29]. B6 deficiency leads to uracil incorporation and chromosome breaks which makes organism susceptible to cancer [30]. Lower vitamin B6 status is also associated with pediatric AML [31]. Combining our KEGG results, we infer that vitamin B6 supplement may have favorable effect on prognosis of pediatric AML patients. Another important pathway is Hippo signaling pathway, which shows dual functions in tumorigenesis according to previous studies [32–34] and its role in pediatric AML remains to be detected. Three miRNA target genes also include NRAS, RUNX1, MAPK1, which are precisely enriched in acute myeloid leukemia pathway. Moreover, prion diseases pathway is also find significant in our analysis. Previous data suggest that prion protein participates in human leukocyte maturation [35] and the expression level of prion protein mRNA can be down-regulated in all-trans retinoic acid induced maturation in NB4 cells [36].

However, the limitations of our study should be considered for future applications. First, the number of pediatric and adolescent CN-AML patients enrolled in our study was only 59, which indicates our sample size is not large enough to draw a reliable conclusion, although we carried out permutation test and LOO-CV—validation techniques for small samples. Second, the number of pediatric and adolescent AML patients who were cytogenetically normal were relatively small and we did not find a suitable external dataset to validate our formula in previous study. External datasets are needed for evaluation of the 3-miRNA signature.

In conclusion, we identified a 3-miRNA signature through genome-wide miRNA expression profiling from TCGA, which could be used as an independent indicator for prognosis of pediatric and adolescent CN-AML patients.

## MATERIALS AND METHODS

### TCGA AML dataset

Expression levels of miRNA and clinical information of patients with AML were downloaded from The Cancer Genome Atlas (TCGA). Level 3 data of miRNA expression level includes 1539 miRNAs and clinical dataset contained 491 patients. Patients were screened by the following criteria. First, patients without miRNA information were excluded. Second, patients who were not cytogenetically normal or cytogenetically information unknown were removed. Third, patients who were alive and the last contact days were unavailable were discarded. Finally, 59 CN-AML patients who were under 21 at diagnosis were included in our study.

Overall survival times (OS) and disease free survival times (DFS) are considered as endpoints respectively. As the data was extracted from TCGA, an ethics committee was not needed.

### Cell culture and transfection

U937 cells were obtained from ATCC. Cells were maintained in RPMI 1640 medium containing 10% fetal bovine serum (FBS, Gibco). Cells were transfected with hsa-miR-181c mimics and negative control (NC) using lipofectamine 3000 (Thermo). Hsa-miR-181c mimics was purchased from GenePharma.

### Statistical analysis

The expression level of 1539 miRNAs was presented as reads per million (RPM) miRNA mapped data and the miRNAs whose expression level equal 0 RPM in more than 25% of all observations were eliminated using R (version 3.3.2). Each miRNA was put into univariate Cox's model (miRNA was transformed into binary variable), *P value* and FDR adjustment were used to select miRNAs which are significantly associated with OS or DFS. Significant parameters were filtered out using 0.05 as the cutoff in both *P value* and FDR adjustment. Seventeen miRNAs were identified as biomarker of outcome. Clinical information including gender, race, white blood cell counts, and molecular mutations reported to be associated with prognosis (FLT3-ITD, NPM1, CEBPA) were also used to build univariate Cox's model under the same standard. Each significant miRNA identified by univariate proportional hazards regression was further evaluated in multivariate Cox's model (backward stepwise). To determine whether the miRNA signature can independently predict the prognosis, multivariate analysis was carried out using molecular mutations associated with prognosis and the risk score constructed by miRNA signature.

Permutation test was used to estimate whether the 3-miRNA expression signature can precisely predict OS in population of pediatric and adolescent CN-AML patients by R Bioconductor. It is used to evaluate the performance and randomness of the model. In brief, we take the combination of overall survival time and vital status of patients in the research as a label, then each individual in our study has a label and a risk score which is calculated using the proposed 3-miRNA scoring system. A random system was constructed by assigned labels randomly to individuals while the risk score keeps consistent with each individual. The random system was tested for survival significance. If the model performs well, a random system cannot predict the prognosis of patients. The area under Receiver Operating Characteristic curve (AUC) was supposed to equal 0.5. A thousand random systems were created by R, after all iterations, significance between AUC of random systems and the right label system was measured by *P-value* with a cutoff 0.05. *P-value* calculated greater than 0.05 means the 3-miRNA signatures have no effect on the outcome.

Leave-one-out Cross Validation (LOO-CV) was also applied to evaluate the model proposed above using R Bioconductor. LOO-CV is powerful in estimating a model's performance. Briefly, each time a observation was left out and all the other observations were used to construct a model using 3 miRNAs above and then makes a prediction for the observation left. 59 tests were conducted and the average AUC was carried out.

### Bioinformatic analysis

We use TargetScan, miRanda and miRTarBase to find target genes of three miRNAs. Gene ontology (GO) analysis was carried out by The Database for Annotation, Visualization and Integrated Discovery (DAVID) online and Kyoto Encyclopedia of Genes and Genomes (KEGG) analysis was carried out by KOBAS online.

### Flow cytometry

Cells were stained with monoclonal antibodies in Annexin V-FITC/PI Apoptosis Detection Kit (BD Bioscience) by protocol. Data were collected using FACSCanto II (BD Bioscience) and were analyzed with FlowJo software (TreeStar, version 7.6.1).

### Cell proliferation assay

Cell proliferation was carried out using the Cell Counting Kit-8 (Dojindo, Kumamoto, Japan) according to the manufacturer's instruction. U937 cells were seeded into 96-well plates at a concentration of 5000–8000 cells/well. Next, 10 ul CCK-8 was added to the cells and the cells were incubated for 2.5 hours at 37°C at each time point. The optical density was read at 450nm with a microplate spectrophotometer.

## SUPPLEMENTARY TABLES







## Abbreviations

AML: Pediatric acute myeloid leukemia
CN-AML: Cytogenetically normal acute myeloid leukemia
ITD allelic ratio: FLT3-ITD mutation to wild-type ratio
TCGA: The Cancer Genome Atlas
OS: Overall Survival Times
DFS: Disease Free Survival Times
AUC: The area under Receiver Operating Characteristic curve
LOO-CV: Leave-one-out Cross Validation
GO: Gene ontology
DAVID: The Database for Annotation, Visualization and Integrated Discovery
KEGG: Kyoto Encyclopedia of Genes and Genomes
