# Supplementary material for: A 3-miRNA signature predicts prognosis of pediatric and adolescent cytogenetically normal acute myeloid leukemia
Source: Oncotarget. 2017 Apr 17;8(24):38902–13. doi: 10.18632/oncotarget.17151 (PMC5503581; doi:10.18632/oncotarget.17151)
Supplement: Supplementary file 1 [file oncotarget-08-38902-s001.pdf]

A 3-miRNA signature predicts prognosis of pediatric and adolescent cytogenetically normal acute myeloid leukemia

Supplementary Materials

Supplementary Table 1: Target genes of three miRNAs. See Supplementary_Table_1

Supplementary Table 2: GO analysis conducted by DAVID. See Supplementary_Table_2
