# Supplementary material for: A 3-miRNA signature predicts prognosis of pediatric and adolescent cytogenetically normal acute myeloid leukemia
Source: Oncotarget. 2017 Apr 17;8(24):38902–13. doi: 10.18632/oncotarget.17151 (PMC5503581; doi:10.18632/oncotarget.17151)
Supplement: Supplementary file 2 [file oncotarget-08-38902-s002.docx]

Table S1. Target genes of three miRNAs

| Mature miRNA | Target |
| --- | --- |
| hsa-mir-146b | ADAM19 |
| hsa-mir-146b | APP |
| hsa-mir-146b | CA6 |
| hsa-mir-146b | FZD1 |
| hsa-mir-146b | GRID1 |
| hsa-mir-146b | KPNA6 |
| hsa-mir-146b | MMP16 |
| hsa-mir-146b | MORN4 |
| hsa-mir-146b | POU3F2 |
| hsa-mir-146b | RNF115 |
| hsa-mir-146b | ROBO1 |
| hsa-mir-146b | SAMD8 |
| hsa-mir-146b | SIAH2 |
| hsa-mir-146b | SP8 |
| hsa-mir-146b | TMEM136 |
| hsa-mir-146b | TTPAL |
| hsa-mir-146b | USP47 |
| hsa-mir-146b | WASF3 |
| hsa-mir-146b | YWHAE |
| hsa-mir-146b | ZNF148 |
| hsa-mir-146b | ZNRF3 |
| hsa-mir-181c | ADAM11 |
| hsa-mir-181c | ADRBK1 |
| hsa-mir-181c | AHNAK |
| hsa-mir-181c | AK3 |
| hsa-mir-181c | AKAP5 |
| hsa-mir-181c | AKIRIN1 |
| hsa-mir-181c | ARF3 |
| hsa-mir-181c | ARL1 |
| hsa-mir-181c | ATMIN |
| hsa-mir-181c | ATP1B1 |
| hsa-mir-181c | ATXN7 |
| hsa-mir-181c | BAG4 |
| hsa-mir-181c | BEND3 |
| hsa-mir-181c | C14orf28 |
| hsa-mir-181c | C16orf87 |
| hsa-mir-181c | C1orf50 |
| hsa-mir-181c | C7orf41 |
| hsa-mir-181c | CBFA2T2 |
| hsa-mir-181c | CD80 |
| hsa-mir-181c | CDH8 |
| hsa-mir-181c | CNNM2 |
| hsa-mir-181c | CNOT1 |
| hsa-mir-181c | COL16A1 |
| hsa-mir-181c | CREB1 |
| hsa-mir-181c | CUL5 |
| hsa-mir-181c | CYR61 |
| hsa-mir-181c | DGCR2 |
| hsa-mir-181c | DNAJC13 |
| hsa-mir-181c | DNAJC21 |
| hsa-mir-181c | E2F7 |
| hsa-mir-181c | ECT2L |
| hsa-mir-181c | EGR1 |
| hsa-mir-181c | ELAVL2 |
| hsa-mir-181c | EMX2 |
| hsa-mir-181c | ERLIN2 |
| hsa-mir-181c | ESD |
| hsa-mir-181c | FAM102A |
| hsa-mir-181c | FAM160A1 |
| hsa-mir-181c | FAM179B |
| hsa-mir-181c | FAM26E |
| hsa-mir-181c | FAM58A |
| hsa-mir-181c | FAM73B |
| hsa-mir-181c | FAM89A |
| hsa-mir-181c | FIGN |
| hsa-mir-181c | FLT1 |
| hsa-mir-181c | FMR1 |
| hsa-mir-181c | FNDC3B |
| hsa-mir-181c | GDPD1 |
| hsa-mir-181c | GHITM |
| hsa-mir-181c | GOLGA1 |
| hsa-mir-181c | GOSR1 |
| hsa-mir-181c | GPSM1 |
| hsa-mir-181c | GRM5 |
| hsa-mir-181c | HMGB2 |
| hsa-mir-181c | HS6ST1 |
| hsa-mir-181c | IL1A |
| hsa-mir-181c | INO80 |
| hsa-mir-181c | ISPD |
| hsa-mir-181c | KAT2B |
| hsa-mir-181c | KCTD10 |
| hsa-mir-181c | KIF3B |
| hsa-mir-181c | KLHL3 |
| hsa-mir-181c | LIF |
| hsa-mir-181c | LIN28A |
| hsa-mir-181c | LMTK2 |
| hsa-mir-181c | LRRC8D |
| hsa-mir-181c | MAPK1 |
| hsa-mir-181c | MAPT |
| hsa-mir-181c | MBOAT1 |
| hsa-mir-181c | MPP7 |
| hsa-mir-181c | NDRG4 |
| hsa-mir-181c | NFATC2IP |
| hsa-mir-181c | NOVA1 |
| hsa-mir-181c | NRAS |
| hsa-mir-181c | OSBPL8 |
| hsa-mir-181c | PAK4 |
| hsa-mir-181c | PBX1 |
| hsa-mir-181c | PDHX |
| hsa-mir-181c | PHF3 |
| hsa-mir-181c | PLCL2 |
| hsa-mir-181c | PRIMA1 |
| hsa-mir-181c | RABGEF1 |
| hsa-mir-181c | RAN |
| hsa-mir-181c | RASSF1 |
| hsa-mir-181c | RASSF6 |
| hsa-mir-181c | RBM27 |
| hsa-mir-181c | RFX2 |
| hsa-mir-181c | RIMKLB |
| hsa-mir-181c | RLF |
| hsa-mir-181c | RPS6KB1 |
| hsa-mir-181c | RUNX1 |
| hsa-mir-181c | SAMD12 |
| hsa-mir-181c | SCML2 |
| hsa-mir-181c | SCN3B |
| hsa-mir-181c | SENP1 |
| hsa-mir-181c | SENP2 |
| hsa-mir-181c | SIK3 |
| hsa-mir-181c | SIPA1L2 |
| hsa-mir-181c | SLA |
| hsa-mir-181c | SLC25A24 |
| hsa-mir-181c | SMAD7 |
| hsa-mir-181c | SNN |
| hsa-mir-181c | SOCS2 |
| hsa-mir-181c | SPP1 |
| hsa-mir-181c | SSBP2 |
| hsa-mir-181c | SSX2IP |
| hsa-mir-181c | TAF9B |
| hsa-mir-181c | TBL1XR1 |
| hsa-mir-181c | TGIF2 |
| hsa-mir-181c | THBS4 |
| hsa-mir-181c | TIAL1 |
| hsa-mir-181c | TMEM181 |
| hsa-mir-181c | TMEM71 |
| hsa-mir-181c | TRAF6 |
| hsa-mir-181c | UNC5A |
| hsa-mir-181c | WASF1 |
| hsa-mir-181c | WHAMM |
| hsa-mir-181c | XRN1 |
| hsa-mir-181c | ZDHHC3 |
| hsa-mir-181c | ZDHHC7 |
| hsa-mir-181c | ZFHX4 |
| hsa-mir-181c | ZFX |
| hsa-mir-181c | ZNF292 |
| hsa-mir-181c | ZNF654 |
| hsa-mir-181c | ZNF667 |
| hsa-mir-181c | ZSCAN12 |
| hsa-mir-181c | ZZZ3 |
| hsa-mir-4786 | ABCF3 |
| hsa-mir-4786 | ACOT13 |
| hsa-mir-4786 | AEN |
| hsa-mir-4786 | AFF2 |
| hsa-mir-4786 | AGXT |
| hsa-mir-4786 | AGXT2 |
| hsa-mir-4786 | AHCYL2 |
| hsa-mir-4786 | ALG10B |
| hsa-mir-4786 | ALG13 |
| hsa-mir-4786 | ALG14 |
| hsa-mir-4786 | AMDHD2 |
| hsa-mir-4786 | AP3S2 |
| hsa-mir-4786 | APOC3 |
| hsa-mir-4786 | ARHGEF18 |
| hsa-mir-4786 | ARSB |
| hsa-mir-4786 | ATP6V0E1 |
| hsa-mir-4786 | ATXN3 |
| hsa-mir-4786 | BICD2 |
| hsa-mir-4786 | BLOC1S5 |
| hsa-mir-4786 | BZW1 |
| hsa-mir-4786 | C14orf180 |
| hsa-mir-4786 | C15orf38-AP3S2 |
| hsa-mir-4786 | C18orf32 |
| hsa-mir-4786 | C9orf3 |
| hsa-mir-4786 | CCDC36 |
| hsa-mir-4786 | CCSER2 |
| hsa-mir-4786 | CEP97 |
| hsa-mir-4786 | CLCC1 |
| hsa-mir-4786 | COX6A1 |
| hsa-mir-4786 | COX6A1P2 |
| hsa-mir-4786 | COX6B1 |
| hsa-mir-4786 | CPT1A |
| hsa-mir-4786 | CRNKL1 |
| hsa-mir-4786 | CTSS |
| hsa-mir-4786 | DCTN6 |
| hsa-mir-4786 | DCUN1D5 |
| hsa-mir-4786 | DNAJC10 |
| hsa-mir-4786 | DOCK11 |
| hsa-mir-4786 | DTWD2 |
| hsa-mir-4786 | DYRK2 |
| hsa-mir-4786 | EHD1 |
| hsa-mir-4786 | ELOVL7 |
| hsa-mir-4786 | ELP3 |
| hsa-mir-4786 | ENAH |
| hsa-mir-4786 | ENPP1 |
| hsa-mir-4786 | EXOSC2 |
| hsa-mir-4786 | FAM118A |
| hsa-mir-4786 | FGG |
| hsa-mir-4786 | FOSL2 |
| hsa-mir-4786 | FPR1 |
| hsa-mir-4786 | FYTTD1 |
| hsa-mir-4786 | GFPT1 |
| hsa-mir-4786 | GGA2 |
| hsa-mir-4786 | GGCX |
| hsa-mir-4786 | GNL3L |
| hsa-mir-4786 | GSG2 |
| hsa-mir-4786 | GSPT1 |
| hsa-mir-4786 | GTF2F1 |
| hsa-mir-4786 | GTF3C6 |
| hsa-mir-4786 | HAUS5 |
| hsa-mir-4786 | HEATR3 |
| hsa-mir-4786 | HOXD3 |
| hsa-mir-4786 | IFNE |
| hsa-mir-4786 | IFNGR2 |
| hsa-mir-4786 | IL17REL |
| hsa-mir-4786 | ITPRIPL2 |
| hsa-mir-4786 | JOSD1 |
| hsa-mir-4786 | KIAA1841 |
| hsa-mir-4786 | KLF2 |
| hsa-mir-4786 | KLHL11 |
| hsa-mir-4786 | KPNA4 |
| hsa-mir-4786 | LDLR |
| hsa-mir-4786 | LHX4 |
| hsa-mir-4786 | LRP3 |
| hsa-mir-4786 | LRTOMT |
| hsa-mir-4786 | LUZP2 |
| hsa-mir-4786 | LZIC |
| hsa-mir-4786 | MAG |
| hsa-mir-4786 | MAGI3 |
| hsa-mir-4786 | MAPKAPK5 |
| hsa-mir-4786 | MAST3 |
| hsa-mir-4786 | MAVS |
| hsa-mir-4786 | MB21D1 |
| hsa-mir-4786 | MCCC2 |
| hsa-mir-4786 | MCUR1 |
| hsa-mir-4786 | MICA |
| hsa-mir-4786 | MMP17 |
| hsa-mir-4786 | MOB1B |
| hsa-mir-4786 | MOB3A |
| hsa-mir-4786 | MRO |
| hsa-mir-4786 | MSMO1 |
| hsa-mir-4786 | MTFMT |
| hsa-mir-4786 | MYO10 |
| hsa-mir-4786 | NBPF12 |
| hsa-mir-4786 | NOA1 |
| hsa-mir-4786 | NRXN3 |
| hsa-mir-4786 | NT5DC3 |
| hsa-mir-4786 | NUDT7 |
| hsa-mir-4786 | NUP205 |
| hsa-mir-4786 | OGFRL1 |
| hsa-mir-4786 | ORAI2 |
| hsa-mir-4786 | ORC6 |
| hsa-mir-4786 | OSBPL2 |
| hsa-mir-4786 | OTUD3 |
| hsa-mir-4786 | PAICS |
| hsa-mir-4786 | PAK2 |
| hsa-mir-4786 | PAPLN |
| hsa-mir-4786 | PAPOLG |
| hsa-mir-4786 | PCDHB11 |
| hsa-mir-4786 | PCYT2 |
| hsa-mir-4786 | PDE11A |
| hsa-mir-4786 | PDE6D |
| hsa-mir-4786 | PDXK |
| hsa-mir-4786 | PEX2 |
| hsa-mir-4786 | PEX26 |
| hsa-mir-4786 | PHACTR4 |
| hsa-mir-4786 | PHAX |
| hsa-mir-4786 | PLEKHG4B |
| hsa-mir-4786 | PM20D2 |
| hsa-mir-4786 | PNPO |
| hsa-mir-4786 | POLI |
| hsa-mir-4786 | PRKAR2A |
| hsa-mir-4786 | PRR12 |
| hsa-mir-4786 | PTGES3L |
| hsa-mir-4786 | PURB |
| hsa-mir-4786 | QPCTL |
| hsa-mir-4786 | RAB3GAP1 |
| hsa-mir-4786 | RAD51 |
| hsa-mir-4786 | RBM28 |
| hsa-mir-4786 | RNF24 |
| hsa-mir-4786 | RRP8 |
| hsa-mir-4786 | S1PR2 |
| hsa-mir-4786 | SF3B3 |
| hsa-mir-4786 | SF3B3 |
| hsa-mir-4786 | SFR1 |
| hsa-mir-4786 | SFT2D2 |
| hsa-mir-4786 | SH3RF2 |
| hsa-mir-4786 | SLC10A6 |
| hsa-mir-4786 | SLC1A4 |
| hsa-mir-4786 | SLC25A34 |
| hsa-mir-4786 | SLC7A6OS |
| hsa-mir-4786 | SLFN5 |
| hsa-mir-4786 | STX6 |
| hsa-mir-4786 | TBC1D15 |
| hsa-mir-4786 | TBC1D19 |
| hsa-mir-4786 | TCEA3 |
| hsa-mir-4786 | TCEB3 |
| hsa-mir-4786 | TFDP2 |
| hsa-mir-4786 | TLE3 |
| hsa-mir-4786 | TMEM170B |
| hsa-mir-4786 | TMEM59 |
| hsa-mir-4786 | TNFRSF10B |
| hsa-mir-4786 | TRIM17 |
| hsa-mir-4786 | TRIM45 |
| hsa-mir-4786 | UBE2B |
| hsa-mir-4786 | UBE2D3 |
| hsa-mir-4786 | URGCP |
| hsa-mir-4786 | USP6NL |
| hsa-mir-4786 | VPS8 |
| hsa-mir-4786 | WDR13 |
| hsa-mir-4786 | WDR77 |
| hsa-mir-4786 | WEE1 |
| hsa-mir-4786 | YTHDC1 |
| hsa-mir-4786 | ZC3HAV1 |
| hsa-mir-4786 | ZDHHC8 |
| hsa-mir-4786 | ZNF264 |
| hsa-mir-4786 | ZNF273 |
| hsa-mir-4786 | ZNF280B |
| hsa-mir-4786 | ZNF324B |
| hsa-mir-4786 | ZNF431 |
| hsa-mir-4786 | ZNF460 |
| hsa-mir-4786 | ZNF468 |
| hsa-mir-4786 | ZNF486 |
| hsa-mir-4786 | ZNF554 |
| hsa-mir-4786 | ZNF573 |
| hsa-mir-4786 | ZNF652 |
| hsa-mir-4786 | ZNF714 |
| hsa-mir-4786 | ZSCAN22 |
| hsa-mir-4786 | ZSWIM7 |
