# Supplementary material for: A 3-miRNA signature predicts prognosis of pediatric and adolescent cytogenetically normal acute myeloid leukemia
Source: Oncotarget. 2017 Apr 17;8(24):38902–13. doi: 10.18632/oncotarget.17151 (PMC5503581; doi:10.18632/oncotarget.17151)
Supplement: Supplementary file 3 [file oncotarget-08-38902-s003.docx]

Table S2. GO analysis conducted by DAVID

| Category | ID | Term | Count | P |
| --- | --- | --- | --- | --- |
| Biological process | 0045449 | regulation of transcription | 64 | 0.002 |
| Biological process | 0006355 | regulation of transcription, DNA-dependent | 47 | 0.003 |
| Biological process | 0051252 | regulation of RNA metabolic process | 47 | 0.004 |
| Biological process | 0045664 | regulation of neuron differentiation | 8 | 0.008 |
| Biological process | 0006350 | transcription | 51 | 0.010 |
| Biological process | 0010769 | regulation of cell morphogenesis involved in differentiation | 6 | 0.011 |
| Biological process | 0050770 | regulation of axonogenesis | 5 | 0.017 |
| Biological process | 0046907 | intracellular transport | 20 | 0.019 |
| Biological process | 0070647 | protein modification by small protein conjugation or removal | 8 | 0.021 |
| Biological process | 0008219 | cell death | 21 | 0.024 |
| Biological process | 0050767 | regulation of neurogenesis | 8 | 0.025 |
| Biological process | 0016265 | death | 21 | 0.025 |
| Biological process | 0060284 | regulation of cell development | 9 | 0.026 |
| Biological process | 0006886 | intracellular protein transport | 13 | 0.029 |
| Biological process | 0010975 | regulation of neuron projection development | 5 | 0.032 |
| Biological process | 0006605 | protein targeting | 9 | 0.033 |
| Biological process | 0045596 | negative regulation of cell differentiation | 9 | 0.034 |
| Biological process | 0007268 | synaptic transmission | 11 | 0.034 |
| Biological process | 0010557 | positive regulation of macromolecule biosynthetic process | 19 | 0.034 |
| Biological process | 0016926 | protein desumoylation | 2 | 0.034 |
| Biological process | 0006928 | cell motion | 15 | 0.035 |
| Biological process | 0019226 | transmission of nerve impulse | 12 | 0.040 |
| Biological process | 0008154 | actin polymerization or depolymerization | 3 | 0.042 |
| Biological process | 0009719 | response to endogenous stimulus | 13 | 0.048 |
| Biological process | 0051960 | regulation of nervous system development | 8 | 0.048 |
| Biological process | 0048545 | response to steroid hormone stimulus | 8 | 0.048 |
| Biological process | 0000375 | RNA splicing, via transesterification reactions | 7 | 0.049 |
| Biological process | 0000377 | RNA splicing, via transesterification reactions | 7 | 0.049 |
| Biological process | 0000398 | nuclear mRNA splicing, via spliceosome | 7 | 0.049 |
| Biological process | 0045927 | positive regulation of growth | 5 | 0.049 |
| Biological process | 0031328 | positive regulation of cellular biosynthetic process | 19 | 0.049 |
| Biological process | 0010563 | negative regulation of phosphorus metabolic process | 4 | 0.050 |
| Biological process | 0045936 | negative regulation of phosphate metabolic process | 4 | 0.050 |
| Cellular components | 0031974 | membrane-enclosed lumen | 49 | 0.000 |
| Cellular components | 0043233 | organelle lumen | 48 | 0.000 |
| Cellular components | 0031981 | nuclear lumen | 40 | 0.000 |
| Cellular components | 0070013 | intracellular organelle lumen | 45 | 0.000 |
| Cellular components | 0005654 | nucleoplasm | 26 | 0.002 |
| Cellular components | 0044451 | nucleoplasm part | 18 | 0.004 |
| Cellular components | 0005667 | transcription factor complex | 9 | 0.014 |
| Cellular components | 0005730 | nucleolus | 19 | 0.018 |
| Cellular components | 0005643 | nuclear pore | 5 | 0.031 |
| Cellular components | 0005802 | trans-Golgi network | 4 | 0.032 |
| Molecular function | 0008270 | zinc ion binding | 61 | 0.002 |
| Molecular function | 0003677 | DNA binding | 58 | 0.010 |
| Molecular function | 0046914 | transition metal ion binding | 65 | 0.022 |
| Molecular function | 0030528 | transcription regulator activity | 39 | 0.024 |
| Molecular function | 0003690 | double-stranded DNA binding | 6 | 0.032 |
| Molecular function | 0043566 | structure-specific DNA binding | 7 | 0.049 |
